# Comparison of Anthropometric Equations to Estimate Body Composition in Chilean Male Adolescent Soccer Players

**DOI:** 10.3390/jfmk11030326

**Published:** 2026-08-22

**Authors:** Álvaro Farfán-Díaz, Miguel Alarcón-Rivera, Marcelo Andrade Oyarzun, Daniel Tapia-Villanueva, Exal Garcia-Carrillo, Iván Molina-Márquez, Rodrigo Yáñez-Sepúlveda, Dario Barrera-González, Felipe Montalva-Valenzuela

**Affiliations:** 1 Carrera de Nutrición y Dietética, Facultad de Ciencias de la Salud, Universidad de Tarapacá, Arica 1000000, Chile; 2 Escuela de Ciencias del Deporte y Actividad Física, Facultad de Salud, Universidad Santo Tomás, Talca 3460000, Chile; mrivera3@santotomas.cl; 3 Departamento de Nutrición y Dietética, Universidad de Magallanes, Punta Arenas 6210427, Chile; marcelo.andrade2491@gmail.com; 4 Centro Asistencial de Docencia e Investigación, Universidad de Magallanes (CADI-UMAG), Punta Arenas 6210427, Chile; 5 Independent Researcher, Santiago 8320000, Chile; sportsciencelover@gmail.com; 6 Department of Physical Activity Sciences, Faculty of Education Sciences, Universidad Católica del Maule, Talca 3480112, Chile; 7 Department of Physical Activity Sciences, Universidad de Los Lagos, Osorno 5290000, Chile; 8 Pedagogía en Educación Física, Facultad de Educación, Universidad Adventista de Chile, Chillán 3780000, Chile; ivanmolina@unach.cl; 9 Programa Doctorado en Ciencias de la Actividad Física, Universidad Católica del Maule, Talca 3460000, Chile; 10 Escuela de Ciencias del Deporte, Facultad de Educación y Humanidades, Universidad Andres Bello, Viña del Mar 2200055, Chile; rodrigo.yanez.s@unab.cl; 11 School of Medicine, Universidad Espíritu Santo, Samborondón 092301, Ecuador; 12 Carrera de Kinesiología, Facultad de Ciencias de la Salud, Universidad Autónoma de Chile, Talca 3460000, Chile; dario.barrera@cloud.uautonoma.cl; 13 Escuela de Entrenador en Actividad Física y Deporte, Facultad de Ciencias Humanas, Universidad Bernardo O’Higgins, Santiago 8370993, Chile; felipe.montalva@ubo.cl

**Keywords:** kinanthropometry, body composition, adiposity, youth soccer, skeletal muscle, method comparison

## Abstract

**Background**: Anthropometric body-composition assessment in youth soccer commonly relies on equations developed in different source populations and based on distinct body-composition models, potentially altering nutritional and sport-related interpretation. This study compared body-fat estimates from equations intended to report the same outcome, body fat percentage, but based on different operational models, and quantified muscle-mass outputs from anthropometric approaches targeting total-body skeletal, whole-body, and tissue-level muscle compartments in a cohort of Chilean male U-15 and U-16 players. **Methods**: This secondary cross-sectional method-comparison study analyzed anonymized anthropometric records collected in 2018 from 50 male players. Slaughter, Faulkner, Carter, Deurenberg, and Sloan–Siri estimates of body fat were compared, together with Poortmans, Lee, Doupe, and Kerr muscle-mass approaches. Absolute agreement among adiposity equations was evaluated using a two-way random-effects, absolute-agreement, single-measure ICC [ICC(A,1)] and Bland–Altman analyses. Muscle-mass approaches were compared with repeated-measures analysis; pairwise difference-versus-mean summaries across different muscle constructs were interpreted descriptively rather than as formal agreement tests. Sensitivity analyses excluded Deurenberg and examined a maturation-related Slaughter scenario. **Results**: Deurenberg yielded the highest body-fat estimate (18.97 ± 3.07%), followed by Slaughter (13.32 ± 3.35%), Faulkner (10.84 ± 1.40%), Sloan-Siri (8.01 ± 2.80%), and Carter (7.91 ± 1.49%). Global differences were large (χ^2^(4) = 182.96; *p* < 0.001; Kendall’s W = 0.915), and individual absolute agreement was low [ICC(A,1) = 0.157; bootstrap 95% CI 0.102–0.212]. Excluding Deurenberg did not remove the between-method differences (χ^2^(3) = 124.44; *p* < 0.001; W = 0.830). For muscle mass, mean estimates ranged from 25.11 ± 3.07 kg with Poortmans to 31.26 ± 4.94 kg with Doupe, with a significant method effect after Greenhouse–Geisser correction (*p* < 0.001). **Conclusions**: The adiposity equations yielded substantially different numerical estimates of the same reported outcome, body fat percentage, and showed low individual-level absolute agreement. Muscle-mass approaches also produced large numerical differences, partly expected because they target different compartments. Outputs from different equations or constructs should not be treated as numerically interchangeable. Because no criterion reference method was included, this study quantifies method dependence rather than validity and cannot identify the most accurate equation.

## 1. Introduction

Body composition refers to the quantitative study of the main components of the human body, their relationships, and their changes in response to biological, nutritional, and functional factors [1,2]. The field is commonly organized across atomic, molecular, cellular, tissue-system, and whole-body levels [1]. From this perspective, terms used in body-composition assessment do not necessarily describe the same component: fat mass belongs to the molecular level, adipose tissue to the tissue level, and fat-free mass is not equivalent to skeletal muscle mass [1,2]. Fat-free mass comprises water, protein, minerals, and other non-fat constituents, whereas skeletal muscle mass represents a tissue-system component; in growing populations such as adolescents, changes in hydration and mineralization can alter fat-free mass without a proportional change in skeletal muscle mass [3].

These conceptual distinctions have direct methodological implications. Two-compartment models divide the body into fat mass and fat-free mass, assuming relatively constant density, hydration, and mineral content of fat-free mass; these assumptions may be less stable in children and adolescents because of growth, maturation, and tissue-distribution changes [3,4,5]. Multicomponent models reduce part of this error by separating additional components, whereas the Kerr five-component anthropometric model fractionates body mass into skin, adipose tissue, muscle, bone, and residual tissue [6,7]. Although this five-component fractionation offers a more detailed anatomical breakdown, simpler skinfold- and girth-based equations remain widely used in applied and field settings because they require less equipment and specialized training; this justifies examining how much these more accessible field-based approaches diverge from one another in practice [8,9,10].

In sport science and sports nutrition, body-composition assessment is used to describe an athlete’s morphological profile, monitor training adaptations, and contextualize nutritional decisions. For youth players, interpretation requires particular attention because anthropometric profiles vary with age, biological maturation, playing position, and training characteristics [11,12,13,14,15].

In practice, anthropometric approaches developed in different source populations and under different assumptions are often used concurrently. Some estimate body fat percentage from skinfolds, such as Slaughter, Faulkner, and Carter; others use general variables, such as Deurenberg, or estimate body density before conversion to body fat percentage, as in Sloan [16,17,18,19,20,21]. Although all five approaches are intended to report body fat percentage, they do not operationalize adiposity identically. They differ in developmental sample, age range, predictor set, reference method, and underlying body-composition assumptions. Kerr adipose tissue was not included in the present adiposity agreement analysis because it represents a tissue-level component rather than a molecular-level fat-mass estimate [1,6,7]. Such differences are plausible mechanisms for systematic numerical disagreement when the equations are applied to the same trained adolescent cohort.

Beyond adiposity, muscle mass is functionally relevant during adolescent development because of its relationship with force production, acceleration, power, and training adaptation [13,14,15]. Yet the available anthropometric approaches target related but distinct constructs: Poortmans and Lee estimate total-body skeletal muscle mass, Doupe estimates whole-body muscle mass in males, and Kerr estimates tissue-level muscle mass within a five-component model [6,7,22,23,24]. These outputs arise from different body-composition frameworks and should not be treated as numerically interchangeable or as directly equivalent to a single criterion-defined skeletal-muscle compartment [1].

Comparisons with dual-energy X-ray absorptiometry (DXA) reinforce the importance of this distinction. In youth soccer, relevant differences between anthropometric approaches and criterion methods have been documented, and several field procedures have shown bias that limits direct numerical comparability [25,26,27,28,29]. For muscle mass, González-Mendoza et al. [30] reported different levels of agreement with DXA across anthropometric approaches in professional male players.

Therefore, this study compared body fat percentage estimates and examined anthropometric approaches to muscle-mass estimation in a cohort of Chilean male U-15 and U-16 players, explicitly distinguishing total-body skeletal muscle mass, whole-body muscle mass, and tissue-level muscle mass. For adiposity, the objective was to evaluate numerical differences and individual-level agreement among equations intended to report body fat percentage, while recognizing differences in their operational models and assumptions. For muscle mass, the objective was to quantify the magnitude and direction of outputs from approaches targeting related but distinct compartments rather than to test construct equivalence. This distinction is practically relevant because values produced by different equations may be compared during longitudinal monitoring, against reference data, or across studies and services. We hypothesized that adiposity equations developed in different source populations and using different predictors would yield systematically different estimates and limited absolute agreement. For muscle mass, we expected systematic numerical differences because the target compartments differ, with the broader whole-body muscle output represented by the Doupe calculation expected to be higher than skeletal-muscle estimates; no fixed directional ordering was specified for Kerr because it represents a distinct tissue-fractionation model.

## 2. Materials and Methods

### 2.1. Study Design and Conceptual Approach

This study was a secondary cross-sectional analysis of previously collected anthropometric records, using a within-subject method-comparison design. The same measurement battery was used to generate estimates of adiposity and muscle mass with several published approaches. The coded and anonymized records were collected in 2018 during the undergraduate thesis project titled “Relationship between Dietary Intake and Body Composition in Semi-Professional Adolescent Soccer Players from Club San Marcos de Arica” [31], conducted at Universidad de Tarapacá. The thesis was academically approved and archived in the Universidad de Tarapacá Library System under institutional record No. 000080190 [31]. The comparative analyses reported here have not previously been published in a peer-reviewed article. The present study applies agreement and sensitivity analyses not used in the original thesis and focuses on the effect of method selection in a Chilean youth-soccer cohort.

The conceptual framework distinguished body-composition levels and models. The method-comparison design quantified differences among approaches commonly used in practice while preserving the distinction among the body compartments they target.

### 2.2. Participants and Ethics

The analytical source set comprised 52 male players from the U-15 and U-16 development categories of a single club linked to the Chilean National Professional Football Association. Participants were selected through convenience sampling based on access to the club and eligibility for the original assessment. One player was excluded because a musculoskeletal injury prevented regular attendance at training, and one was excluded because of repeated training absences. Both exclusions therefore reflected failure to meet the eligibility requirement of regular training attendance, leaving 50 participants for the present analysis. Players were included if they were regularly involved in training and competition and had the anthropometric measurements required for the selected methods.

Given the secondary nature of the study, sample size was determined by the availability of valid records. A sensitivity analysis for paired comparisons was conducted in G*Power version 3.1.9.7 to contextualize the statistical sensitivity of the available sample. With α = 0.05, 80% power, and *n* = 50, the detectable standardized paired difference was approximately *dz* = 0.40, corresponding to a small-to-moderate effect. A conventional medium effect (*dz* = 0.50) would require approximately 34 participants.

The study followed the principles of the Declaration of Helsinki [32]. The original data collection was conducted as part of an undergraduate thesis in Nutrition and Dietetics at Universidad de Tarapacá, Faculty of Health Sciences, Department of Kinesiology and Nutrition, and received documented academic and institutional authorization within the Nutrition and Dietetics Program (NUDIET No. 09.18, 5 March 2018), distinct from the thesis library accession number No. 000080190. The participating sports institution separately authorized the fieldwork, with coordination from the coaching staff. Parents or legal guardians provided written informed consent, and players provided written assent before the original assessments. The parental consent and adolescent assent documentation included explicit optional authorization for coded/anonymized data to be retained and used in subsequent research related to body composition, anthropometry, dietary intake, and nutritional status in athletes. Supporting consent, assent, and institutional documentation had previously been provided to the JFMK Editorial Office during the initial editorial ethics assessment and made available for editorial and reviewer evaluation. For the present manuscript, the authors accessed only a previously coded and anonymized database; no new recruitment, intervention, participant contact, or access to directly identifying personal data was performed.

### 2.3. Anthropometric Procedures

Variables included age, body mass, standing height, sitting height, body mass index (BMI), bone breadths, body girths, and skinfolds. For adiposity, the measurements compatible with the selected equations were used, including triceps, subscapular, supraspinale, abdominal, mid-thigh, and medial-calf skinfolds. Biological maturation was estimated using the somatic maturity-offset equation of Mirwald et al. [33], which uses age, body mass, standing height, sitting height, and leg length to predict years from peak height velocity (PHV). Players were classified as pre-PHV (offset < −1.0 year), circa-PHV (−1.0 to +1.0 year), or post-PHV (offset > +1.0 year).

Data were collected between June and July 2018 in groups organized according to player and coaching-staff availability. Assessments were conducted in a room within the sports complex that was prepared to provide privacy, adequate lighting, and ventilation. The archived documentation does not preserve a standardized fasting protocol, a measured hydration-status criterion, a controlled ambient-temperature value, or sufficiently detailed information on the interval since the most recent training session; these conditions were therefore not reconstructed retrospectively.

Measurements followed the International Standards for Anthropometric Assessment available at the time of data collection in 2011 [8]. A.F.-D., an ISAK Level 2 anthropometrist, was the sole anthropometrist responsible for the specialized anthropometric assessments used in the present analyses. All skinfolds, girths, bone breadths, and segmental measurements were obtained by A.F.-D. Thesis assistants supported basic measurements, participant organization, and data recording under his supervision, but did not perform the specialized ISAK measurements used in the body-composition equations. Before participant assessment, A.F.-D. completed pre-field technical-error exercises documented in the original thesis archive. Recalculation from the archived duplicate measurements yielded intra-evaluator %TEM ranges of 2.19–4.38% for the six skinfolds, 0.32–0.74% for girths, and 0.57–0.67% for bone breadths used in the present analyses. These values were below the ISAK precision thresholds applied to experienced anthropometrists (≤5% for skinfolds and ≤1% for other measurements) [8]. Reliability coefficients ranged from R = 0.890–0.994 for skinfolds, 0.993–0.997 for girths, and 0.911–0.987 for bone breadths. Because A.F.-D. was the sole anthropometrist for these specialized measurements, interobserver TEM was not applicable. The archive does not preserve replicate-level field logs for every final participant measurement, so no unverified third-measurement rule or aggregation procedure was reconstructed. Participants were assessed wearing light sports clothing and barefoot when required.

Standing height and sitting height were measured using a SECA 213 stadiometer (seca GmbH & Co. KG, Hamburg, Germany). Body mass was measured with SECA 803 and 813 scales. Girths were measured with a Cescorf anthropometric tape (Cescorf Equipamentos, Porto Alegre, Brazil); skinfolds with a Slim Guide caliper (Creative Health Products, Ann Arbor, MI, USA); small bone breadths with a Cescorf Innovare anthropometer or small-breadth caliper; large breadths with a Faga long anthropometer or large-breadth caliper (FAGA, Rosario, Argentina); and segment lengths with a Faga metallic segmometer. A 40 × 50 × 30 cm anthropometric box was used to standardize selected positions.

For equations using corrected girths, the corrected value was calculated as follows: corrected girth (cm) = measured girth (cm) − [π × skinfold thickness (cm)]. When skinfolds were recorded in millimeters, they were divided by 10 before applying the correction.

### 2.4. Anthropometric Equations

Seven candidate adiposity approaches were considered. Five were retained (Slaughter, Faulkner, Carter, Deurenberg, and Sloan–Siri), whereas Durnin–Womersley and Jackson–Pollock were excluded because the historical measurement battery did not contain all skinfold sites required for their intended implementations and because both procedures were developed primarily in adult populations. Selection of the retained methods was based on compatibility with the available historical variables and previous use or applicability in pediatric, adolescent, or athletic settings [34,35].

For adiposity, Slaughter, Faulkner, Carter, Deurenberg, and Sloan were applied; Sloan yielded body density, which was converted to body fat percentage using Siri [16,17,18,19,20,21]. Slaughter, Faulkner, Carter, and Sloan use skinfold measurements, whereas Deurenberg estimates body fat from BMI, age, and sex. Deurenberg was retained as an exploratory, skinfold-independent comparator rather than as a preferred equation for trained adolescent soccer players because its source population and BMI-based structure differ from the present cohort. The selected equations and their original contexts of application are summarized in Table 1.

In addition, muscle-mass estimates were calculated using Poortmans, Lee, Doupe, and Kerr, and each output was interpreted according to its target construct [6,7,22,23,24]. For Poortmans, skeletal muscle mass (kg) was calculated as height (m) × [0.0064 × CAG^2^ + 0.0032 × CTG^2^ + 0.0015 × CCG^2^] + 2.56 × sex + 0.136 × age, with sex = 1 for males. For Lee, skeletal muscle mass (kg) was calculated as height (m) × [0.00744 × CAG^2^ + 0.00088 × CTG^2^ + 0.00441 × CCG^2^] + 2.4 × sex − 0.048 × age + race + 7.8; the audited calculation used race = 0, corresponding to the White/Hispanic coding in the original model. In both Poortmans and Lee, CAG was relaxed-arm girth corrected by triceps skinfold, CTG was mid-thigh girth corrected by mid-thigh skinfold, and CCG was maximal-calf girth corrected by medial-calf skinfold. For Doupe, the published regression coefficients were retained: MM (g) = height (cm) × [0.031 × MUThG^2^ + 0.064 × CCG^2^ + 0.089 × CAG^2^] − 3006, with the result divided by 1000 to obtain kilograms [22]. In the archived calculation, CAG was relaxed-arm girth corrected by triceps skinfold, MUThG was maximal-thigh girth corrected by supraspinale skinfold, and CCG was maximal-calf girth corrected by medial-calf skinfold. Kerr was applied as a tissue-level five-component fractionation model. These four approaches were compared to quantify practical method dependence, not to imply construct equivalence. The Martin et al. [36] equation was not included because it was developed and validated in adult populations. The methods, target constructs, and implementation details are summarized in Table 2.

### 2.5. Data Management and Statistical Analysis

Primary statistical analyses were performed in jamovi for macOS, version 2.6.45.0 (The jamovi Project, Sydney, Australia). The historical dataset was organized in Microsoft Excel for Mac, version 16.109 (Microsoft Corporation, Redmond, WA, USA). Independent recalculation, bootstrap confidence intervals, sensitivity analyses, Supplementary Tables, and participant-level agreement plots were generated in Python 3.13.5 using NumPy 2.3.5, SciPy 1.17.0, statsmodels 0.14.6, and Matplotlib 3.10.8.

Before the present secondary analysis, the working database was reviewed for completeness, consistency, and anonymization. The analytic file contained only coded participant identifiers and the anthropometric variables required to calculate the selected equations. Names, contact details, and other direct identifiers were not included. Potential graphical outliers were identified using the conventional boxplot criterion of values beyond 1.5 × the interquartile range; they were retained when source-file auditing showed no transcription error, and the measurement remained physiologically plausible. All inferential analyses therefore used the 50 complete eligible cases.

Continuous variables were described using mean and standard deviation (SD), median, and range as appropriate. Normality was assessed with the Shapiro–Wilk test. Because several adiposity estimates were non-normal and all equations were applied to the same participants, body-fat percentage was compared using the Friedman test, with Kendall’s W as the global effect size, followed by paired Wilcoxon signed-rank tests with Holm adjustment and matched-pairs rank-biserial correlations as pairwise effect sizes. Following methodological auditing requested during peer review, two sensitivity analyses were performed: (1) repeating the adiposity comparison without Deurenberg and (2) a Slaughter scenario applying the −5.5 constant to post-PHV players while retaining −3.4 in circa-PHV players. Exploratory Friedman and pairwise ICC analyses were also repeated separately in the circa-PHV and post-PHV groups.

Because all five adiposity approaches are intended to report body fat percentage, numerical agreement in that reported outcome was assessed with a two-way random-effects, absolute-agreement, single-measure intraclass correlation coefficient [ICC(A,1)], treating participant as the target and equation as the method factor [37,38]. This use of agreement statistics does not imply that the equations share identical predictors, reference methods, or body-composition assumptions. In this notation, A denotes absolute agreement, and 1 denotes the reliability of a single-method estimate. Participant-level bootstrap 95% confidence intervals were calculated with 10,000 resamples for the global ICC (random seed 42) and 5000 resamples for pairwise ICC(A,1) estimates. The average-measures ICC(A,k), where k = 5 adiposity equations, is reported only in Supplementary File S1, Table S1, as a descriptive statistic and is not used for practical interpretation because averaging all five equations is not a usual clinical or field application. Bland–Altman analyses reported mean bias, 95% limits of agreement (LoA), and 95% confidence intervals for the bias and both LoA. Proportional bias was assessed by regressing participant-level pairwise differences on the corresponding pairwise means and testing the regression slope against zero. Difference-versus-mean plots and the distributions of pairwise differences were examined for magnitude-dependent or heteroscedastic behavior; logarithmic difference and ratio analyses were additionally performed as sensitivity analyses. No a priori clinical or practical acceptance limits were defined, so LoA were interpreted according to their magnitude and dispersion rather than classified as acceptable or unacceptable. Body-fat percentage estimates were also expressed as estimated fat mass in kilograms [body mass × (%BF/100)].

For muscle mass, normality was assessed for the raw method-specific estimates, residuals from the within-subject model, and the pairwise within-subject differences used for post hoc comparisons. Differences among methods were analyzed using one-way repeated-measures ANOVA. Mauchly’s test was used to evaluate sphericity, and Greenhouse–Geisser-corrected degrees of freedom were reported when sphericity was violated. Partial η^2^ was used as the global effect size. Post hoc comparisons used paired Student’s *t* tests with Holm adjustment and paired standardized mean differences (dz). Because small departures from normality were identified in the relative-percentage residuals and in two pairwise percentage contrasts, Friedman tests with Kendall’s W and Holm-adjusted paired Wilcoxon tests were performed as nonparametric sensitivity analyses. Pairwise difference-versus-mean summaries used the same bias, LoA, confidence-interval, proportional-bias, and graphical procedures described above. Formal agreement interpretation was restricted to Poortmans versus Lee because both target total-body skeletal muscle mass; pairs involving different muscle compartments were interpreted descriptively as systematic numerical offsets rather than as agreement between identical biological quantities. Statistical significance was set at *p* < 0.05.

## 3. Results

The final sample comprised 50 male players from the U-15 and U-16 categories. Mean age was 15.46 ± 0.57 years, body mass 60.84 ± 7.73 kg, standing height 169.26 ± 5.40 cm, and BMI 21.19 ± 2.04 kg/m^2^ (Table 3). The PHV offset was 1.26 ± 0.57 years (range −0.03 to 2.42); 35 players (70.0%) were post-PHV, and 15 (30.0%) were circa-PHV. The cohort comprised 4 goalkeepers, 21 defenders, 10 forwards, and 15 midfielders. Competitive experience was <1 year in 25 players, 1–2 years in 13, and >2 years in 12. All 50 players trained more than four times per week; 37 reported approximately 1–2 h/day and 13 reported >2 h/day. These variables are presented descriptively and were not used for underpowered inferential comparisons by playing position.

Shapiro–Wilk testing indicated departures from normality for Slaughter (W = 0.949, *p* = 0.031), Faulkner (W = 0.945, *p* = 0.021), Deurenberg (W = 0.942, *p* = 0.017), and Sloan–Siri (W = 0.938, *p* = 0.011), whereas Carter was compatible with normality (W = 0.956, *p* = 0.063). Deurenberg yielded the highest body-fat percentage, followed by Slaughter, Faulkner, Sloan–Siri, and Carter. The global comparison showed substantial differences (χ^2^(4) = 182.96; *p* < 0.001; Kendall’s W = 0.915). Nine of ten Holm-adjusted pairwise comparisons were significant; Carter versus Sloan–Siri was not (adjusted *p* = 0.973). Complete pairwise statistics and effect sizes are provided in Supplementary Table S2.

**Figure 1:**
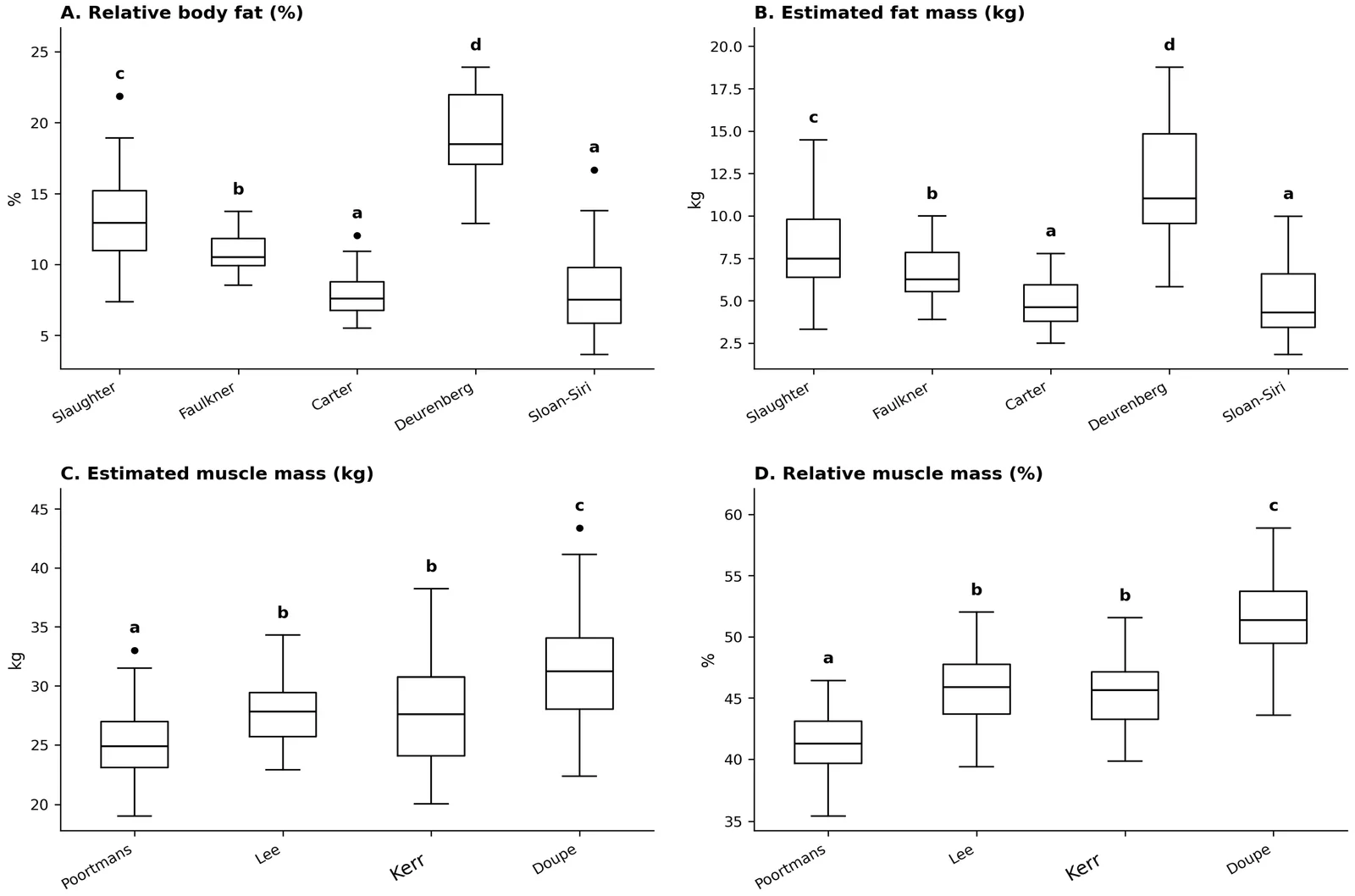
Distribution of anthropometric estimates of body fat and muscle mass according to method. Note: (**A**), body fat percentage; (**B**), estimated fat mass (kg); (**C**), estimated muscle mass (kg); (**D**), relative muscle mass (%). Boxes = interquartile range; central line = median; whiskers = 1.5 × interquartile range. Points beyond 1.5 × IQR were retained after source-file auditing identified no transcription error and the values remained physiologically plausible. Different letters are a visual guide to Holm-adjusted pairwise differences; exact adjusted *p* values and effect sizes are reported in Supplementary Tables S2 and S3. In panels (**C**,**D**), the label Doupe denotes the Doupe implementation described in Section 2.4.

These method-dependent differences are illustrated in Figure 1, which summarizes body fat percentage, estimated fat mass, estimated muscle mass, and relative muscle mass according to the anthropometric method applied.

**Figure 2:**
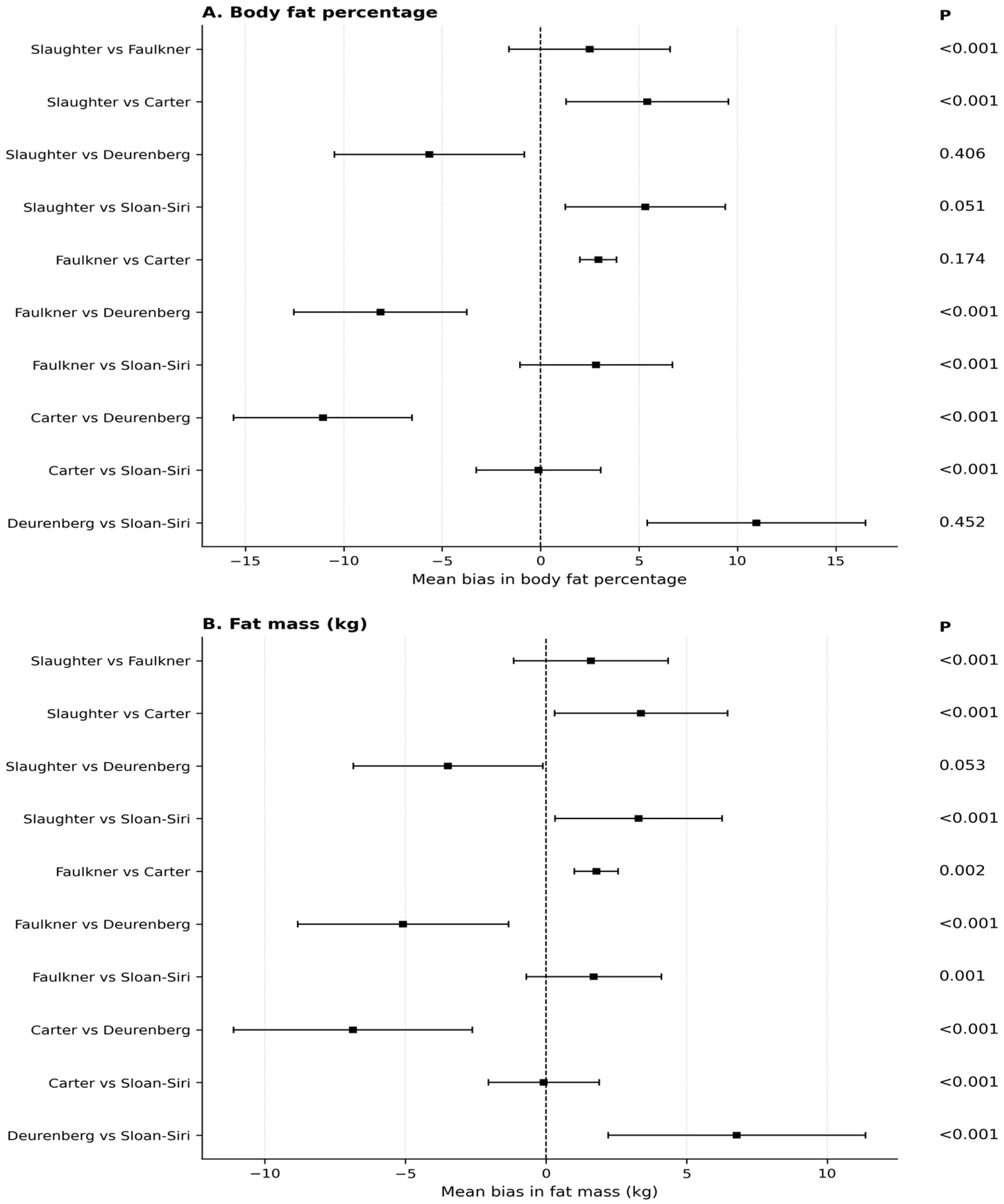
Summary of Bland–Altman mean bias and 95% limits of agreement between anthropometric adiposity methods. Note: Panel (**A**), body fat percentage; Panel (**B**), estimated fat mass (kg). Squares represent mean bias and horizontal bars represent 95% limits of agreement; the vertical dashed line indicates no bias. Complete numerical results are provided in Supplementary Table S4, with participant-level difference-versus-mean plots in Supplementary Figures S1–S3. *p* values correspond to the test of the proportional-bias regression slope.

The global absolute-agreement ICC among the five adiposity equations was low [ICC(A,1) = 0.157; bootstrap 95% CI 0.102–0.212]. Pairwise ICC(A,1) values were strongly method-dependent, ranging from 0.047 for Carter versus Deurenberg to 0.744 for Carter versus Sloan–Siri (Supplementary Table S1). Bland–Altman analyses showed systematic bias and wide LoA. For example, Carter minus Deurenberg yielded a mean bias of −11.06 percentage points (95% CI −11.71 to −10.40), with LoA from −15.59 (95% CI −16.72 to −14.46) to −6.52 (95% CI −7.65 to −5.39). Complete numerical Bland–Altman results are provided in Supplementary Table S4, with participant-level difference-versus-mean plots in Supplementary Figures S1–S3. Log/ratio sensitivity analyses did not materially alter the qualitative interpretation. Because no a priori acceptance limits were defined, these LoA are interpreted by their magnitude and dispersion rather than as categorically acceptable or unacceptable. These adiposity agreement findings are summarized in Figure 2.

Sensitivity analyses did not alter the principal interpretation. After excluding Deurenberg, between-method differences remained large (χ^2^(3) = 124.44; *p* < 0.001; W = 0.830), and the ICC(A,1) among the four skinfold-based methods increased to 0.334 (bootstrap 95% CI 0.241–0.405) but remained low at the individual level. In the maturation-based Slaughter scenario, mean Slaughter %BF decreased from 13.32 ± 3.35% to 11.85 ± 3.24%; when Deurenberg was simultaneously excluded, the remaining four methods still differed (χ^2^(3) = 105.14; *p* < 0.001; W = 0.701). The five-method pattern also persisted when stratified by maturity status: circa-PHV, *n* = 15, χ^2^(4) = 53.81, *p* < 0.001, W = 0.897; post-PHV, *n* = 35, χ^2^(4) = 129.99, *p* < 0.001, W = 0.928. Pairwise ICCs ranged from 0.072 to 0.773 in circa-PHV and 0.031 to 0.709 in post-PHV. These subgroup analyses are exploratory because of the unequal and modest subgroup sizes.

All four raw muscle-mass estimates were compatible with normality in kilograms (all Shapiro–Wilk *p* ≥ 0.371) and relative percentage (all *p* ≥ 0.272). Residuals from the within-subject model were compatible with normality for kilograms (*p* = 0.158), whereas the relative-percentage residuals showed a small departure (*p* = 0.029). All pairwise differences in kilograms were compatible with normality; for relative percentage, Poortmans versus Kerr (*p* = 0.0036) and Poortmans versus Doupe (*p* = 0.026) departed from normality, while the remaining contrasts did not. A nonparametric sensitivity analysis confirmed the same global inference for both kilograms and relative percentage [Friedman χ^2^(3) = 118.824; *p* < 0.001; Kendall’s W = 0.792], and Holm-adjusted Wilcoxon comparisons reproduced the qualitative pairwise pattern, with Lee versus Kerr remaining the only non-significant pair. Poortmans, Lee, Kerr, and Doupe yielded mean estimates of 25.11 ± 3.07, 27.69 ± 2.61, 27.62 ± 4.40, and 31.26 ± 4.94 kg, respectively; the corresponding relative values were 41.38 ± 2.39%, 45.77 ± 2.85%, 45.30 ± 2.96%, and 51.27 ± 3.30% (Table 4). Mauchly’s test indicated violation of sphericity for both outcomes. For muscle mass in kilograms, W = 0.111, χ^2^(5) = 104.91, *p* < 0.001, Greenhouse–Geisser ε = 0.462, and the corrected ANOVA remained significant [F(1.387, 67.963) = 194.11, *p* < 0.001, partial η^2^ = 0.798]. For relative muscle mass, W = 0.232, χ^2^(5) = 69.74, *p* < 0.001, ε = 0.536, with F(1.607, 78.752) = 226.27, *p* < 0.001, partial η^2^ = 0.822. All Holm-adjusted paired *t*-test comparisons were significant except Lee versus Kerr in kilograms (mean difference = 0.066 kg, adjusted *p* = 0.816); complete parametric post hoc results are provided in Supplementary Table S3.

**Figure 3:**
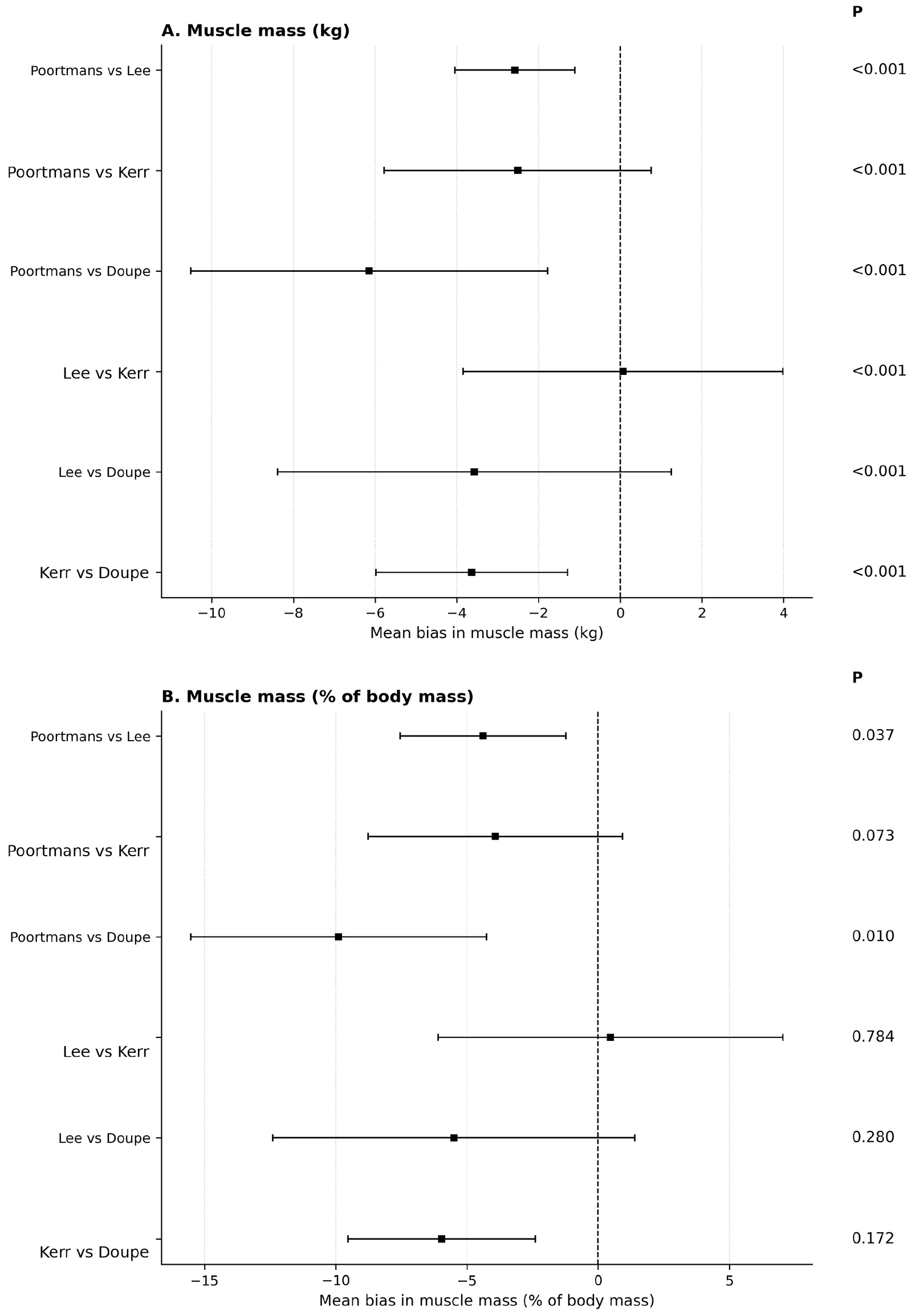
Pairwise difference-versus-mean summary of anthropometric muscle-mass outputs. Note: Panel (**A**), muscle mass (kg); Panel (**B**), relative muscle mass (% of body mass). Squares represent mean pairwise differences and horizontal bars represent the corresponding 95% limits; the vertical dashed line indicates zero difference. For Poortmans versus Lee, which target the same total-body skeletal-muscle construct, bias and limits are interpreted as method-agreement statistics. For pairs involving different muscle compartments, the same numerical summaries are shown descriptively to quantify offset and dispersion and are not interpreted as formal agreement between identical biological quantities. Complete numerical summaries are provided in Supplementary Table S5; participant-level difference-versus-mean plots for the same-construct Poortmans-versus-Lee comparison are provided in Supplementary Figure S4. *p*-values correspond to the test of the proportional-bias regression slope. The figure label Doupe denotes the implementation described in Section 2.4.

Pairwise difference-versus-mean summaries quantified method-dependent offsets in muscle-mass outputs (Figure 3). Formal Bland–Altman agreement interpretation was restricted to Poortmans versus Lee because both approaches target total-body skeletal muscle mass. For this comparison, the mean Poortmans-Lee bias was −2.58 kg (95% CI −2.79 to −2.36), with LoA from −4.04 to −1.11 kg, and the proportional-bias slope was 0.162 (*p* < 0.001). For cross-construct pairs, the same numerical summaries are reported descriptively to quantify offset and dispersion rather than formal agreement. The largest offset involved Poortmans versus Doupe: −6.15 kg (95% CI −6.78 to −5.51), with LoA from −10.51 kg (95% CI −11.60 to −9.42) to −1.78 kg (95% CI −2.87 to −0.69). This direction is expected in part because Poortmans targets skeletal muscle mass whereas the Doupe calculation represents a broader whole-body muscle construct. Lee and Kerr had a near-zero mean difference in kilograms (0.07 kg; 95% CI −0.50 to 0.63), but this numerical proximity does not imply construct identity or individual equivalence. For Kerr quality control, the ratio of the sum of the five estimated tissue components to measured body mass averaged 0.999996 (99.9996%), with a range of 0.999824–1.000000; the maximum absolute closure error was 0.0135 kg. Complete numerical pairwise summaries are provided in Supplementary Table S5; participant-level difference-versus-mean plots for the same-construct Poortmans-versus-Lee comparison are provided in Supplementary Figure S4.

## 4. Discussion

This study demonstrated substantial method-dependent divergence among commonly used anthropometric approaches in a cohort of Chilean male U-15 and U-16 soccer players. For adiposity, all five equations report body fat percentage but operationalize this outcome through different predictors, reference methods, source populations, and body-composition assumptions; the large differences, low ICC(A,1), and wide pairwise LoA therefore indicate limited individual-level numerical agreement in the reported outcome. For muscle mass, the approaches target related but distinct compartments, so part of the numerical divergence is expected a priori and should be interpreted as construct-specific offset rather than measurement disagreement between identical quantities. The adiposity pattern remained strong after excluding Deurenberg under a maturation-based Slaughter sensitivity scenario and within both the circa-PHV and post-PHV subgroups. Overall, the findings show that source population, predictor set, measurement sites, reference methods, and target construct materially influence the numerical estimate obtained from the same athletes [1,16,17,18,19,20,21,22,23,24,39,40].

For adiposity, the present mean values can be contextualized against Chilean and international youth-soccer evidence. García et al. [25] reported DXA body fat of 12.9 ± 4.1% in 43 young Chilean elite players (16.9 ± 1.3 years), together with Faulkner 11.1 ± 1.6% and a Slaughter implementation of 11.5 ± 3.2%; the corresponding values in the present cohort were 10.84 ± 1.40% and 13.32 ± 3.35%. Direct numerical comparison must be approached with caution because the studies did not use identical Slaughter implementations or measurement protocols. In adolescent football players, Lozano-Berges et al. [26] likewise found that DXA, air-displacement plethysmography, bioimpedance, and Slaughter estimates were not interchangeable, with wide limits of agreement. Their later validation study [27] found significant bias in six published anthropometric equations against DXA and showed better performance for a football-specific equation. These external data support population- and equation-specific interpretation rather than assuming that a commonly used formula is universally valid.

Adolescence adds complexity because growth, mineralization, biological maturation, and tissue distribution may modify the relationship between skinfolds, girths, and total body composition [3,4,5,41,42]. Importantly, PHV offset and Tanner stage are related but non-equivalent maturational constructs. The Slaughter sensitivity analysis therefore should not be interpreted as a retrospective Tanner classification; it demonstrates only that the principal method-dependence result persists under a plausible alternative constant. Likewise, the persistence of disagreement between circa-PHV and post-PHV subgroups suggests that somatic maturity did not eliminate between-method differences, although these subgroup estimates are exploratory because of limited sample size.

For muscle mass, construct and implementation distinctions are essential. Poortmans and Lee estimate total-body skeletal muscle mass, the Doupe equation targets whole-body muscle mass in males, and Kerr provides a tissue-level muscle component [6,7,22,23,24]. In the present cohort, Doupe yielded the highest relative estimate (51.27 ± 3.30% of body mass). This value should not be interpreted as directly equivalent to a DXA-defined skeletal-muscle compartment. In 179 professional male soccer players, González-Mendoza et al. [30] reported DXA-derived skeletal muscle mass of 32.4 ± 3.5 kg; Lee showed the best agreement with DXA (mean difference +1.10 kg; LoA −1.83 to 4.03 kg), whereas Doupe averaged 38.7 ± 5.1 kg and overestimated DXA by 6.31 kg. Kerr also yielded higher values than DXA in that adult cohort. These findings support cautious, construct-specific interpretation and reinforce that the higher or lower numerical estimate cannot be assumed to be physiologically more correct. Practically, values generated under one muscle-mass construct should not be substituted for another in longitudinal tracking, reference-value interpretation, or cross-study comparison; the exact equation and target construct should be reported explicitly [43].

### 4.1. Strengths and Limitations

The principal strengths are the within-subject design, the simultaneous application of several commonly used field approaches to the same measurement battery, and the explicit separation of skeletal-muscle, whole-body muscle, and tissue-level constructs. This design allows the numerical consequences of method selection to be quantified without attributing the observed method-dependent divergence to differences between participant samples.

Several limitations should be acknowledged. First, no criterion-reference method such as DXA or a multicomponent model was available, so the study cannot determine which equation is most accurate. Second, the convenience sample was relatively small, male only, and drawn from a single youth club; field-position comparisons were therefore not pursued inferentially. Third, biological maturation was estimated from a predictive PHV equation rather than a criterion maturational assessment. Fourth, although pre-field intra-evaluator %TEM and reliability data were recovered for A.F.-D., replicate-level field logs for every final participant measurement and pre-field quality-control records for every variable used in every equation were not available. Interobserver TEM was not applicable to the specialized ISAK measurements because A.F.-D. was the sole anthropometrist responsible for those assessments. Fifth, skinfolds were measured with a Slim Guide caliper, whereas several source equations were developed with other caliper models; differences in spring pressure and jaw characteristics may contribute to systematic measurement variation. Sixth, the data were collected in 2018 and analyzed secondarily in 2026, and fasting status, hydration status, room temperature, and precise recovery time since the previous training session were not recoverable from the archive. Seventh, detailed individual ancestry/ethnicity data compatible with the coding used in the Lee equation were not collected; race = 0 was used because this is the White/Hispanic coding in the original model, without implying a homogeneous racial classification of Chilean participants. Finally, the Doupe calculation used the anthropometric measurements available in the historical dataset: relaxed-arm girth corrected by triceps skinfold for CAG, maximal-thigh girth corrected by supraspinale skinfold for MUThG, and maximal-calf girth corrected by medial-calf skinfold for CCG. This same operationalization has been described previously in Chilean rugby and soccer athletes [44], while other applications of Doupe in professional soccer have used a different thigh operationalization [30]. This site-specific variability should be considered when comparing absolute Doupe estimates across studies.

### 4.2. Future Lines of Investigation

Future research should compare these anthropometric approaches directly against criterion methods such as DXA and, when feasible, multicomponent body-composition models in adolescent athletes. Larger samples should include females, multiple clubs and competitive levels, balanced maturational strata, and standardized reporting of fasting, hydration, recent training, environmental conditions, caliper model, and technical error. Longitudinal studies are also needed to determine whether the same equations provide stable tracking of within-athlete change across growth and training seasons.

### 4.3. Practical Recommendations

The practical implication is not that one equation should replace all others, but that method selection should be purposeful and consistent. For adiposity, Deurenberg should be interpreted cautiously in muscular youth athletes because it is BMI-based and was not developed specifically for trained soccer players. External criterion-method studies show that the performance of skinfold equations varies across cohorts; where a population-specific equation has been validated, and the required measurement sites are available, that evidence should guide selection [25,26,27,28]. If a generic field equation is used for monitoring, the same measurement protocol, anatomical sites, caliper, and equation should be retained longitudinally. This consistency reduces the possibility that observed longitudinal differences arise from a change in the estimation method, but it cannot ensure that an observed change is biological because measurement error, growth, maturation, training, and nutritional status also contribute. Values obtained with different equations should not be directly interchanged.

For skeletal muscle, external evidence in professional male soccer players supports Lee as a comparatively strong option against DXA [30], but Lee remains adult-derived and includes an ethnicity term that requires transparent coding. Poortmans has the advantage of having been developed in children and adolescents [24]. The Doupe estimate and Kerr should be interpreted according to their distinct whole-body muscle and tissue-level constructs rather than ranked directly against skeletal-muscle equations. In the absence of a criterion method in the present cohort, these recommendations are conditional on external validation evidence and should not be interpreted as validation of any equation by the current study.

## 5. Conclusions

In this cohort of Chilean male U-15 and U-16 soccer players, the adiposity equations produced large, systematic differences in estimates of the same reported outcome, body fat percentage, and showed low individual-level absolute agreement. This pattern persisted after excluding the BMI-based Deurenberg equation, under a maturation-based Slaughter sensitivity scenario, and within circa-PHV and post-PHV subgroups. Muscle-mass approaches also produced substantial numerical offsets, but these must be interpreted according to their target compartments: Poortmans and Lee estimate total-body skeletal muscle mass, the Doupe approach represents whole-body muscle mass, and Kerr estimates tissue-level muscle mass. Cross-construct offsets are therefore expected and should not be interpreted as formal disagreement between identical biological quantities.

These results support transparent reporting and consistent longitudinal use of the same anthropometric protocol and equation, with interpretation tied to the source population and body-composition construct represented by each method. Because no criterion reference method was available, the study evaluates method dependence rather than criterion validity and cannot establish which equation is most accurate for these players. Criterion-based validation in larger, more diverse youth-soccer cohorts is required before method-specific accuracy recommendations can be made.

## Figures and Tables

**Table 1:** Anthropometric equations used to estimate body fat percentage.

Equation	Expression Applied	Original Context or Interpretation
Slaughter [20]	If ΣSF ≤ 35 mm: %BF = 1.21 × ΣSF − 0.008 × ΣSF^2^ − 3.4; if ΣSF > 35 mm: %BF = 0.783 × ΣSF + 1.6, where ΣSF = triceps + subscapular skinfolds (mm).	Children and youth; coefficients vary by sex, race/ethnicity, and maturational category in the original framework.
Faulkner [18]	%BF = 0.153 × (triceps + subscapular + supraspinale + abdominal; all mm) + 5.783.	Equation widely used in sport settings.
Carter [16]	%BF = 0.1051 × (triceps + subscapular + supraspinale + abdominal + mid-thigh + medial calf; all mm) + 2.585.	Derived from athletic populations.
Deurenberg [17]	%BF = 1.51 × BMI (kg/m^2^) − 0.70 × age (years) − 2.2.	General pediatric BMI-based equation; retained here only as an exploratory comparator.
Sloan [21] + Siri [19]	BD = 1.1043 − (0.001327 × mid-thigh skinfold, mm) − (0.00131 × subscapular skinfold, mm). Then: %BF = (495/BD) − 450.	Body density converted to %BF using Siri.

Note: %BF, body fat percentage; BD, body density; BMI, body mass index; ΣSF, sum of the specified skinfolds. Tanner stage was not collected. The submitted analysis therefore retained the historical pubertal-male Slaughter constant (−3.4) as the primary calculation; a maturation-based sensitivity scenario was additionally examined using −5.5 for players classified as post-PHV. PHV status was not treated as equivalent to Tanner stage.

**Table 2:** Methods used for the muscle mass analysis.

Method	Estimated Construct	Implementation and Interpretation in This Study
Poortmans [24]	Total-body skeletal muscle mass.	Pediatric/adolescent equation; corrected relaxed-arm/triceps, mid-thigh/mid-thigh, and maximal-calf/medial-calf girths; sex = 1 (male).
Lee [23]	Total-body skeletal muscle mass.	Adult-derived exploratory equation; same corrected girths as Poortmans; sex = 1 and race coefficient = 0 (White/Hispanic coding in the original model).
Doupe [22]	Whole-body muscle mass in males.	Published Doupe regression coefficients were retained. CAG used relaxed-arm/triceps, MUThG maximal-thigh/supraspinale, and CCG maximal-calf/medial-calf. See Section 2.4 for the full calculation.
Kerr [6,7]	Tissue-level muscle mass within a five-component model.	Tissue-level five-component model; numerical closure against measured body mass was checked as a quality-control step.

**Table 3:** Descriptive characteristics of the sample.

Variable	*n*	Mean ± SD	Median	Min–Max
Age (years)	50	15.46 ± 0.57	15.54	14.28–16.26
Body mass (kg)	50	60.84 ± 7.73	61.00	45.20–78.50
Standing height (cm)	50	169.26 ± 5.40	170.00	157.80–180.30
BMI (kg/m^2^)	50	21.19 ± 2.04	21.03	17.35–24.56
PHV offset (years)	50	1.26 ± 0.57	1.28	−0.03–2.42
Circa-PHV	15 (30.0%)	—	—	—
Post-PHV	35 (70.0%)	—	—	—

Note: BMI, body mass index; PHV, peak height velocity; SD, standard deviation.

**Table 4:** Descriptive muscle-mass estimates according to anthropometric method.

Method	Mean ± SD (kg)	Median (kg)	Range (kg)	Mean ± SD (%)	Median; Range (%)
Poortmans	25.11 ± 3.07	24.89	19.04–33.01	41.38 ± 2.39	41.30; 35.43–46.43
Lee	27.69 ± 2.61	27.84	22.90–34.30	45.77 ± 2.85	45.91; 39.40–52.02
Kerr	27.62 ± 4.40	27.60	20.05–38.24	45.30 ± 2.96	45.64; 39.85–51.56
Doupe	31.26 ± 4.94	31.25	22.36–43.36	51.27 ± 3.30	51.38; 43.60–58.87

Note: Kerr represents tissue-level muscle mass within the five-component model; Poortmans and Lee estimate skeletal muscle mass; the Doupe estimate represents whole-body muscle mass as calculated from the implementation specified in Section 2.4.

## Data Availability

The anonymized data supporting the findings of this study may be made available from the corresponding author upon reasonable request, subject to ethical and institutional restrictions for secondary use of data from minors. The original thesis from which the anonymized database derives is archived in the Universidad de Tarapacá Library System under institutional record No. 000080190 [31].

## Supplementary Materials

The following supporting information can be downloaded at: https://www.mdpi.com/article/10.3390/jfmk11030326/s1. Supplementary File S1: contains the statistical details requested during peer review.

## Abbreviations

The following abbreviations are used in this manuscript:

%BF	Body fat percentage
BD	Body density
ANOVA	Analysis of variance
PHV	Peak height velocity
BMI	Body mass index
DXA	Dual-energy X-ray absorptiometry
ICC	Intraclass correlation coefficient
ISAK	International Society for the Advancement of Kinanthropometry
SD	Standard deviation
