# Supplementary material for: Comparison of Anthropometric Equations to Estimate Body Composition in Chilean Male Adolescent Soccer Players
Source: J Funct Morphol Kinesiol. 2026 Aug 22;11(3):326. doi: 10.3390/jfmk11030326 (PMC13511747; doi:10.3390/jfmk11030326)
Supplement: Supplementary file 1 [file jfmk-11-00326-s001.zip › Supplementary File S1.pdf]

Supplementary File

Statistical details requested during peer review

Comparison of Anthropometric Equations to Estimate Body Composition in a Cohort of Chilean Male Adolescent Soccer Players

Scope. This reduced supplement contains only information that directly addresses explicit reviewer requests and is too detailed for the main manuscript: pairwise ICCs, complete post hoc comparisons, complete numerical difference-versus-mean summaries, and participant-level difference-versus-mean plots for the primary body-fat-percentage agreement analysis and the Poortmans-versus-Lee same-construct muscle-mass comparison. TEM, sphericity, maturation sensitivity, normality, and nonparametric sensitivity results are reported directly in the main manuscript and are not duplicated here.

Table S1. Pairwise absolute-agreement ICC(A,1) for body-fat percentage estimates.

Comparison	ICC(A,1)	Bootstrap 95% CI
Slaughter vs. Faulkner	0.457	0.377–0.530
Slaughter vs. Carter	0.211	0.148–0.267
Slaughter vs. Deurenberg	0.278	0.176–0.384
Slaughter vs. Sloan-Siri	0.313	0.199–0.413
Faulkner vs. Carter	0.311	0.221–0.383
Faulkner vs. Deurenberg	0.082	0.054–0.113
Faulkner vs. Sloan-Siri	0.334	0.212–0.439
Carter vs. Deurenberg	0.047	0.029–0.067
Carter vs. Sloan-Siri	0.744	0.665–0.797
Deurenberg vs. Sloan-Siri	0.068	0.033–0.106

Note. Two-way random-effects, absolute-agreement, single-measure ICC. Pairwise confidence intervals were obtained by participant-level bootstrap resampling. The global ICC(A,1) and its confidence interval are reported in the main manuscript.

Table S2. Complete pairwise post hoc comparisons for body-fat percentage.

Comparison	Mean diff. (pp)	Median diff. (pp)	Wilcoxon W	Raw p	Holm p	Rank-biserial r
Slaughter vs. Faulkner	2.487	2.036	24.0	<0.001	<0.001	0.962
Slaughter vs. Carter	5.413	4.892	0.0	<0.001	<0.001	1.000
Slaughter vs. Deurenberg	-5.643	-5.471	0.0	<0.001	<0.001	-1.000
Slaughter vs. Sloan-Siri	5.311	5.387	0.0	<0.001	<0.001	1.000
Faulkner vs. Carter	2.926	3.048	0.0	<0.001	<0.001	1.000
Faulkner vs. Deurenberg	-8.131	-8.093	0.0	<0.001	<0.001	-1.000
Faulkner vs. Sloan-Siri	2.824	3.441	46.0	<0.001	<0.001	0.928
Carter vs. Deurenberg	-11.057	-10.869	0.0	<0.001	<0.001	-1.000
Carter vs. Sloan-Siri	-0.103	0.366	634.0	0.973	0.973	0.005
Deurenberg vs. Sloan-Siri	10.954	10.750	0.0	<0.001	<0.001	1.000

Note. Difference is first method minus second method. Holm-adjusted p values and matched-pairs rank-biserial effect sizes are shown.

Table S3. Complete pairwise post hoc comparisons for estimated muscle mass.

Outcome	Comparison	Mean diff. (kg)	95% CI	t	Raw p	Holm p	Paired d_z
kg	Poortmans vs. Lee	-2.576	-2.789–-2.363	-24.330	<0.001	<0.001	-3.441
kg	Poortmans vs. Kerr	-2.510	-2.984–-2.036	-10.645	<0.001	<0.001	-1.505
kg	Poortmans vs. Doupe-derived	-6.147	-6.780–-5.514	-19.515	<0.001	<0.001	-2.760
kg	Lee vs. Kerr	0.066	-0.501–0.633	0.234	0.816	0.816	0.033
kg	Lee vs. Doupe-derived	-3.571	-4.269–-2.872	-10.272	<0.001	<0.001	-1.453
kg	Kerr vs. Doupe-derived	-3.637	-3.977–-3.297	-21.489	<0.001	<0.001	-3.039
% body mass	Poortmans vs. Lee	-4.389	-4.846–-3.931	-19.277	<0.001	<0.001	-2.726
% body mass	Poortmans vs. Kerr	-3.924	-4.626–-3.222	-11.235	<0.001	<0.001	-1.589
% body mass	Poortmans vs. Doupe-derived	-9.888	-10.705–-9.072	-24.332	<0.001	<0.001	-3.441
% body mass	Lee vs. Kerr	0.465	-0.487–1.417	0.981	0.331	0.331	0.139
% body mass	Lee vs. Doupe-derived	-5.500	-6.499–-4.500	-11.055	<0.001	<0.001	-1.563
% body mass	Kerr vs. Doupe-derived	-5.964	-6.481–-5.447	-23.187	<0.001	<0.001	-3.279

Note. Kilogram and relative-percentage analyses are combined to avoid redundant tables. Difference is first method minus second method. Paired dz is the standardized mean difference for paired observations.

Table S4. Complete difference-versus-mean summaries for adiposity outcomes.

Outcome	Comparison	Bias	Bias 95% CI	Lower LoA	Lower LoA 95% CI	Upper LoA	Upper LoA 95% CI	Slope	Slope p
Body fat %	Slaughter vs. Faulkner	2.487	1.894 to 3.080	-1.603	-2.624 to -0.583	6.578	5.557 to 7.598	0.841	<0.001
Body fat %	Slaughter vs. Carter	5.413	4.816 to 6.011	1.291	0.263 to 2.319	9.536	8.507 to 10.564	0.800	<0.001
Body fat %	Slaughter vs. Deurenberg	-5.643	-6.343 to -4.943	-10.470	-11.674 to -9.266	-0.817	-2.021 to 0.387	0.100	0.406
Body fat %	Slaughter vs. Sloan-Siri	5.311	4.722 to 5.899	1.252	0.240 to 2.265	9.369	8.357 to 10.382	0.198	0.051
Body fat %	Faulkner vs. Carter	2.926	2.791 to 3.062	1.991	1.757 to 2.224	3.862	3.628 to 4.095	-0.065	0.174
Body fat %	Faulkner vs. Deurenberg	-8.131	-8.768 to -7.494	-12.523	-13.619 to -11.427	-3.738	-4.834 to -2.642	-0.842	<0.001
Body fat %	Faulkner vs. Sloan-Siri	2.824	2.262 to 3.385	-1.047	-2.012 to -0.081	6.694	5.728 to 7.659	-0.750	<0.001
Body fat %	Carter vs. Deurenberg	-11.057	-11.714 to -10.399	-15.591	-16.722 to -14.460	-6.523	-7.654 to -5.392	-0.803	<0.001
Body fat %	Carter vs. Sloan-Siri	-0.103	-0.562 to 0.357	-3.269	-4.059 to -2.479	3.064	2.274 to 3.854	-0.642	<0.001
Body fat %	Deurenberg vs. Sloan-Siri	10.954	10.150 to 11.758	5.411	4.028 to 6.794	16.498	15.115 to 17.881	0.119	0.452
Fat mass (kg)	Slaughter vs. Faulkner	1.593	1.196 to 1.990	-1.147	-1.830 to -0.463	4.333	3.649 to 5.016	0.595	<0.001
Fat mass (kg)	Slaughter vs. Carter	3.375	2.929 to 3.820	0.303	-0.463 to 1.069	6.447	5.680 to 7.213	0.709	<0.001
Fat mass (kg)	Slaughter vs. Deurenberg	-3.491	-3.980 to -3.003	-6.860	-7.700 to -6.019	-0.123	-0.963 to 0.717	-0.165	0.053
Fat mass (kg)	Slaughter vs. Sloan-Siri	3.288	2.857 to 3.719	0.315	-0.427 to 1.057	6.261	5.520 to 7.003	0.328	<0.001
Fat mass (kg)	Faulkner vs. Carter	1.782	1.670 to 1.894	1.008	0.815 to 1.201	2.556	2.363 to 2.749	0.121	0.002

Fat mass (kg)	Faulkner vs. Deurenberg	-5.084	-5.628 to -4.540	-8.834	-9.769 to -7.898	-1.334	-2.270 to -0.399	-0.744	<0.001
Fat mass (kg)	Faulkner vs. Sloan-Siri	1.695	1.347 to 2.043	-0.706	-1.304 to -0.107	4.096	3.497 to 4.695	-0.324	0.001
Fat mass (kg)	Carter vs. Deurenberg	-6.866	-7.482 to -6.250	-11.111	-12.171 to -10.052	-2.621	-3.680 to -1.561	-0.870	<0.001
Fat mass (kg)	Carter vs. Sloan-Siri	-0.087	-0.372 to 0.199	-2.056	-2.547 to -1.565	1.882	1.391 to 2.374	-0.429	<0.001
Fat mass (kg)	Deurenberg vs. Sloan-Siri	6.779	6.116 to 7.442	2.206	1.065 to 3.347	11.353	10.212 to 12.494	0.527	<0.001

Note. Bias is first method minus second method. LoA, 95% limits of agreement. Slope and p correspond to regression of participant-level differences on pairwise means. Estimated fat mass is included numerically because it is shown in Figure 2 of the main manuscript, but separate participant-level fat-mass plots are not duplicated because fat mass is derived from body-fat percentage and body mass.

Table S5. Pairwise difference-versus-mean summaries for muscle-mass outputs.

Outcome	Comparison	Bias	Bias 95% CI	Lower LoA	Lower LoA 95% CI	Upper LoA	Upper LoA 95% CI	Slope	Slope p
kg	Poortmans vs. Lee	-2.576	-2.789–-2.363	-4.043	-4.410–-3.677	-1.109	-1.475–-0.743	0.162	<0.001
kg	Poortmans vs. Kerr	-2.510	-2.984–-2.036	-5.778	-6.593–-4.963	0.758	-0.057–1.573	-0.364	<0.001
kg	Poortmans vs. Doupe-derived	-6.147	-6.780–-5.514	-10.512	-11.601–-9.423	-1.781	-2.870–-0.692	-0.478	<0.001
kg	Lee vs. Kerr	0.066	-0.501–0.633	-3.845	-4.821–-2.870	3.977	3.002–4.953	-0.518	<0.001
kg	Lee vs. Doupe-derived	-3.571	-4.269–-2.872	-8.388	-9.590–-7.186	1.247	0.045–2.449	-0.623	<0.001
kg	Kerr vs. Doupe-derived	-3.637	-3.977–-3.297	-5.982	-6.567–-5.397	-1.291	-1.876–-0.706	-0.117	<0.001
% body mass	Poortmans vs. Lee	-4.389	-4.846–-3.931	-7.544	-8.331–-6.757	-1.234	-2.021–-0.446	-0.190	0.037
% body mass	Poortmans vs. Kerr	-3.924	-4.626–-3.222	-8.765	-9.972–-7.557	0.917	-0.291–2.124	-0.264	0.073
% body mass	Poortmans vs. Doupe-derived	-9.888	-10.705–-9.072	-15.521	-16.926–-14.116	-4.256	-5.661–-2.851	-0.413	0.010
% body mass	Lee vs. Kerr	0.465	-0.487–1.417	-6.103	-7.741–-4.464	7.032	5.394–8.671	-0.056	0.784
% body mass	Lee vs. Doupe-derived	-5.500	-6.499–-4.500	-12.394	-14.114–-10.674	1.395	-0.325–3.115	-0.216	0.280
% body mass	Kerr vs. Doupe-derived	-5.964	-6.481–-5.447	-9.529	-10.419–-8.640	-2.399	-3.289–-1.510	-0.119	0.172

Note. Formal Bland-Altman agreement interpretation is restricted to Poortmans versus Lee because both estimate total-body skeletal muscle mass. Pairs involving Kerr or Doupe are retained as descriptive numerical offsets only.

Supplementary figure

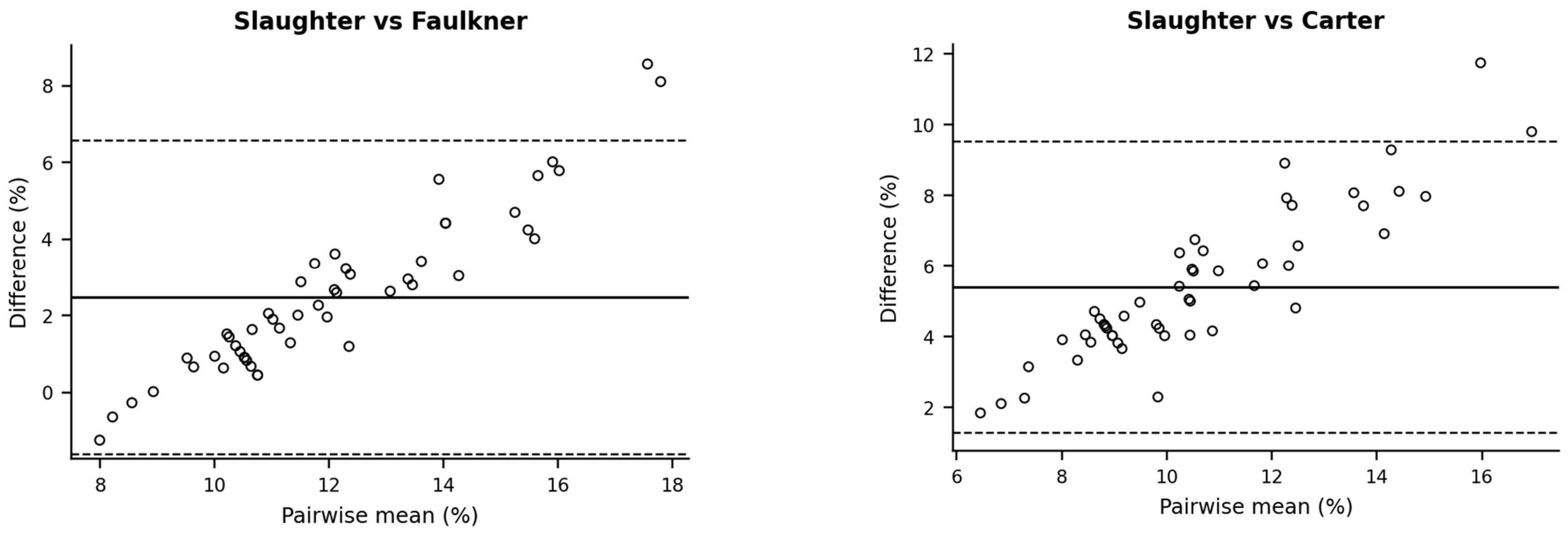

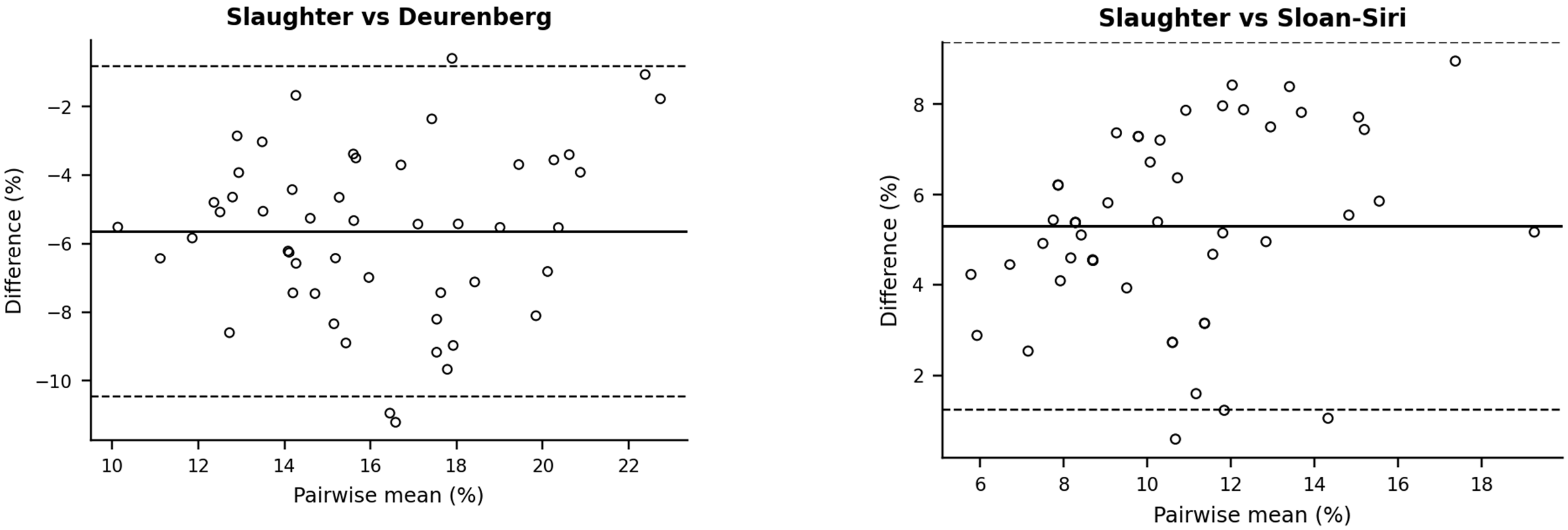

Figure S1. Participant-level difference-versus-mean plots for body-fat percentage comparisons (part 1).

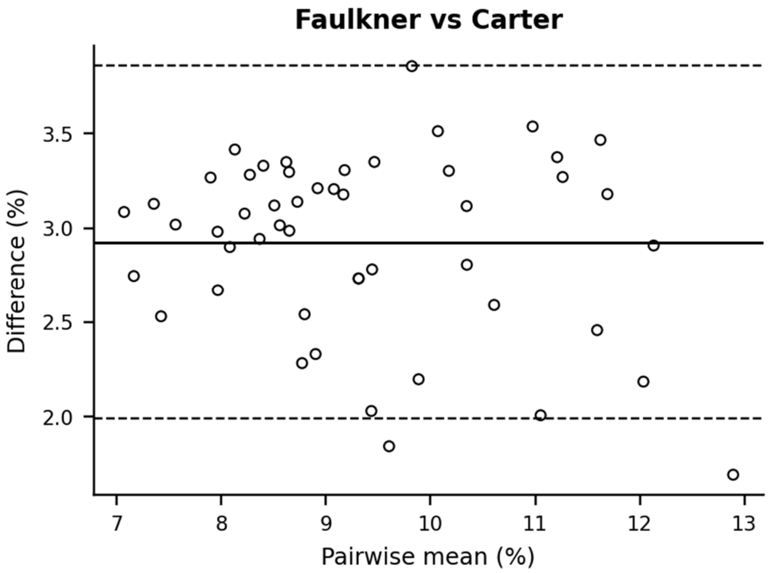

Faulkner vs Carter
Difference (%)
3.5
3.0
2.5
2.0
7
8
9
10
11
12
13
Pairwise mean (%)

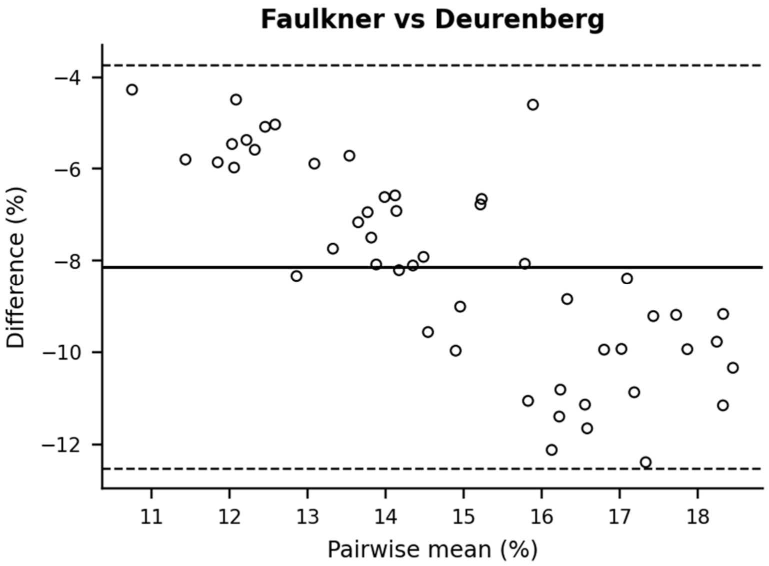

Faulkner vs Deurenberg
Difference (%)
−4
−6
−8
−10
−12
11
12
13
14
15
16
17
18
Pairwise mean (%)

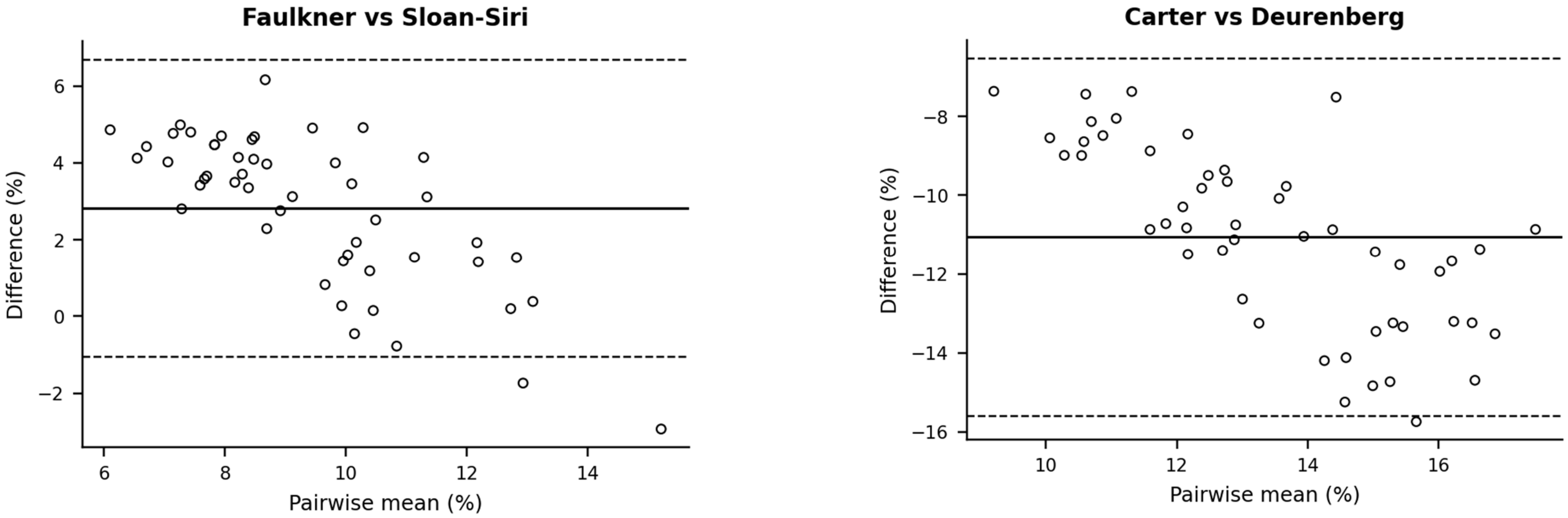

Figure S2. Participant-level difference-versus-mean plots for body-fat percentage comparisons (part 2).

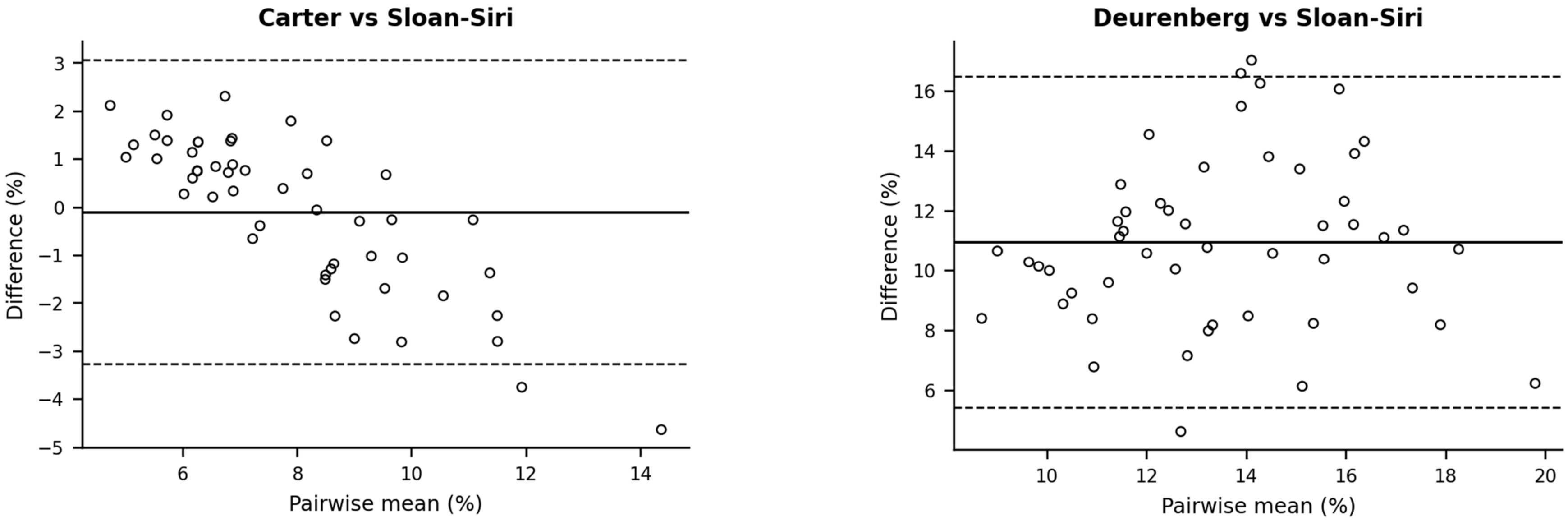

Figure S3. Participant-level difference-versus-mean plots for body-fat percentage comparisons (part 3).

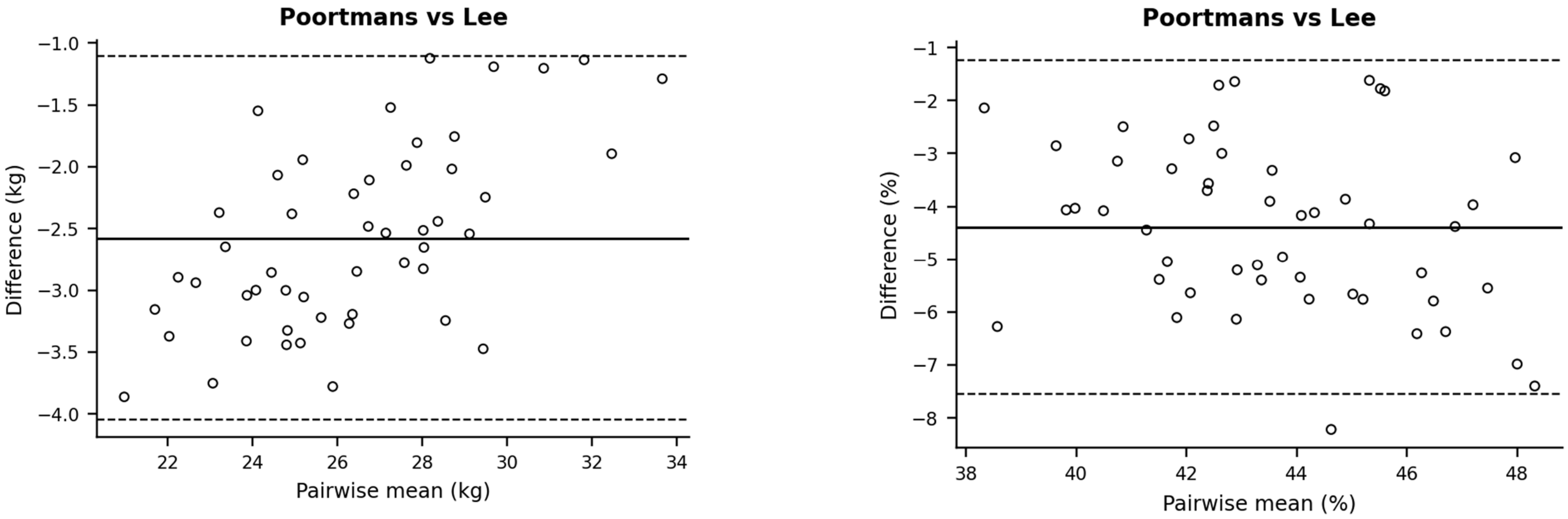

Figure S4. Participant-level difference-versus-mean plots for Poortmans versus Lee skeletal-muscle estimates. Note. The central solid line indicates mean bias and the dashed lines indicate the 95% limits of agreement. Participant-level plots for cross-construct muscle-mass pairs are not presented as formal agreement plots; their numerical offsets are reported in Table S5 and summarized in Figure 3 of the main manuscript.
